# Factors Associated with 30-day Unplanned Readmissions of Sepsis Patients: A Retrospective Analysis of Patients Admitted with Sepsis at a Community Hospital

**DOI:** 10.7759/cureus.5118

**Published:** 2019-07-10

**Authors:** Aditi Singh, Milind Bhagat, Susan V George, Ramya Gorthi, Chandrakanth Chaturvedula

**Affiliations:** 1 Internal Medicine, St. Vincent Hospital, Worcester, USA; 2 Internal Medicine, Rhode Island Hospital, Providence, USA

**Keywords:** sepsis, 30 day readmission, predictors of readmission

## Abstract

Introduction

Mortality from sepsis is decreasing in recent years owing to improved quality of care, targeted programs, and the implementation of sepsis bundles. This has led to an increased pool of sepsis survivors at risk of readmissions. Studies have shown that these sepsis readmissions are common and expensive. The factors associated with these readmissions remain elusive and have incited a lot of research in recent years. The 30-day sepsis readmission rate is increasingly being used as a quality metric for hospitals. A conducted a retrospective chart review analysis of patients admitted with sepsis to find factors affecting the 30-day readmissions of sepsis survivors.

Methods

Patients admitted to our hospital either on the medical-surgical floor or in the intensive care unit (ICU) with an administrative coding for sepsis between January 2014 to November 2015 were identified. A literature search, as well as expert opinion, was considered for the list of factors to be studied, including age, sex, residence on admission, length of stay, getting hemodialysis, hospitalization in the prior year, presence of acute kidney injury (AKI), source of sepsis, discharge disposition, receipt of red blood cell (RBC) products, and route of antibiotics on discharge. A univariate binary logistic regression analysis was performed to test the association between the above-mentioned variables and sepsis readmission. Variables with statistical significance in the univariate analysis were used to compute the multivariate regression analysis along, with adjusted OR and their 95% CI.

Results

A total of 1297 patients were identified with sepsis. Of these, 1068 patients met inclusion criteria. The readmission rate in our study population was 19.19%, and 52% of the readmissions were secondary to an infectious cause. After controlling for the effect of all the potential confounders, the factors that showed a positive association with readmissions were hospitalizations in the year prior to the index hospitalization and discharge to either nursing home or short-term rehab. The requirement of the intensive care unit was not associated with increased readmission. High hemoglobin on discharge was associated with a reduced chance of readmission.

Conclusions

Readmissions after sepsis hospitalization are common and mostly caused by infections. Several factors associated with index sepsis hospitalization can be associated with readmissions. Some of these factors are modifiable and more research is needed to see if these readmissions can be prevented.

## Introduction

Sepsis continues to be a major public health problem in the United States. It is one of the most common causes of hospital admissions, accounting for a major portion of the healthcare expenditure of the country. According to Angus et al., severe sepsis accounted for over 750,000 hospital admissions in 1995, of which 383,000 received intensive care. Since then, various analyses have shown an increasing incidence of sepsis in the US population [1-2].

Over the last several decades, significant progress in understanding the sepsis pathophysiology, earlier recognition, and goal-directed therapies have led to improved outcomes in terms of sepsis mortality and increased number of sepsis survivors [3-4].

These sepsis survivors are often left with significant cognitive and physical disabilities, decreased quality of life, and increased morbidity, leading to an ongoing and long-term requirement for acute care. This causes a significant burden on the health care system [5-7].

Sepsis is one of the top 10 reasons for hospitalization in the United States and is increasingly becoming a common reason for readmissions [8-9]. The US spends around 24.3 billion dollars every year on sepsis-related hospitalization and around 2 billion on readmissions [10]. In an analysis of the 2013 Nationwide Readmissions Database by Mayr et al., sepsis formed a major proportion of unplanned 30-day readmissions as compared to the currently monitored four conditions [11]. These unplanned readmissions also had a longer mean length of stay and higher mean cost per admission.

In recent years, many studies have established increased readmission rates among patients admitted and treated for sepsis [12-13]. However, there is a paucity of studies identifying the reasons or factors associated with these readmissions. By identifying such factors associated with the index sepsis hospitalization, we can focus on the reduction and, ultimately, prevention of readmissions.

To identify some of the missing links, we conducted a retrospective study on patients admitted with sepsis at a community hospital, to identify factors associated with 30-day readmissions. In addition, we hypothesized that patients admitted to the intensive care unit would pose an increased risk of readmissions.

## Materials and methods

Study design

We performed a retrospective cohort study of adults hospitalized with sepsis or severe sepsis at Saint Vincent Hospital, Worcester - a community-based hospital - from January 2014 to November 2015. MetroWest Medical Center Institutional Review Board approved the study with an informed consent exemption.

Outcomes

The primary objective of the study was to determine the rate of 30-day readmissions in patients surviving sepsis in our hospital. The secondary objective was to identify the index hospitalization factors associated with readmission in sepsis patients.

Data collection

Adult patients (age>18) who were hospitalized with sepsis and severe sepsis were included in the study. Both medical and surgical patients were included in the study. Patients who were pregnant, discharged within 24 hours, died during the index hospitalization, transitioned to hospice, or transferred to an outside hospital were excluded from the study. A list of study patients was identified using the International Classification of Diseases, ninth revision, Clinical Modification (ICD-9-CM) codes. The diagnosis was confirmed by a chart review in the electronic medical record (EMR). The international consensus conference definition was used to define sepsis.

Sepsis

Two or more of the following conditions, along with a suspected or confirmed source of infection:

1) Temperature >38°C or <36°C; 2) Heart rate >90 beats per minute; 3) Respiratory rate >20 breaths per minute or PaCO_2_ <32 mmHg; 4) white blood cell (WBC) >12,000/cu mm, <4,000/cu mm, or >10% immature (band) forms.

Severe Sepsis

Sepsis associated with organ dysfunction, hypoperfusion, or hypotension. Hypoperfusion may include but is not limited to lactic acidosis, oliguria, or change in mental status.

Septic Shock

Sepsis-induced hypotension despite adequate fluid resuscitation requiring the use of inotropic or vasopressor agents.

The survivor cohort was analyzed for 30 days after the discharge. For each index hospitalization, patient-level characteristics (age, gender, comorbidities) and hospitalization data, including length of stay, source of infection, red blood cell transfusion, hemoglobin level, white blood cell (WBC) count, presence of renal failure, requirement of hemodialysis, discharge disposition, source of infection, route of antibiotics at discharge, and cause of readmission were collected. These factors were derived based on the combination of a review of previous positive and negative studies and expert opinion.

Statistical analysis

Readmission was considered as the outcome variable. Age, gender, residence at admission, length of stay, hemodialysis, prior hospitalization in one year, peak WBC, presence of AKI, source of infection, discharge disposition, ICU requirement, cause of readmission, duration of antibiotics, discharge hemoglobin, Clostridium difficile infection, red blood cell (RBC) transfusions, and antibiotics at discharge were considered explanatory variables.

A descriptive analysis was carried out by the mean and standard deviation for quantitative variables and by the frequency and proportion for categorical variables.

A univariate binary logistic regression analysis was performed to test the association between the explanatory variables and the outcome variables. The unadjusted odds ratio along with 95% CI are presented. Variables with a statistical significance in the univariate analysis were used to compute the multivariate regression analysis. The adjusted odds ratio along with the 95% CI are presented.

p-value <0.05 was considered statistically significant. IBM SPSS version 22 (IBM Corp., Armonk, NY, US) was used for the statistical analysis.

## Results

Enrollment

There were 1297 patients admitted with sepsis during our study period. A total of 157 patients died during the index hospitalization, and 72 either left against medical advice, transferred to another hospital, or transitioned to hospice, and thus were not considered for the analysis. A total of 1068 subjects were included in the final analysis. Of the 1068 subjects at risk for readmissions, 280 were admitted in the ICU and the remaining 788 were admitted on the medical-surgical floor.

The baseline characteristics at index hospitalization are given in Table 1.

**Table 1:** Descriptive analysis of the baseline characteristics of the study population.

Baseline characteristics of the study population at index hospitalizations.	
Clinical factors	N=1068
Age(years)	
<65	480 (44.94%)
>65	588 (55.06%)
Gender	
Male	567 (53.09%)
Female	501 (46.91%)
Admission place	
Home	818 (76.59%)
Nursing Home	203 (19.01%)
Short term rehab	35 (3.38%)
Jail	4 (0.37%)
Homeless	7 (0.66%)
Hospital transfer	1 (0.09%)
Hemodialysis	
Yes	24 (2.25%)
No	1044 (97.75%)
RIFLE	
R	107 (10.02%)
I	82 (7.68%)
F	42 (3.93%)
L	7 (0.66%)
E	14 (1.31%)
N/A	816 (76.4%)
AKI	252 (23.6%)
No AKI	816 (76.4%)
Prior hospitalization	
Yes	479 (44.85%)
No	589 (55.15%)
RBC Transfusion	
Yes	94 (8.8%)
No	974 (91.2%)
C.diff colitis	
Yes	51 (4.78%)
No	1017(95.22%)
Route of antibiotics at Discharge	
Oral	625 (58.52%)
Intravenous	246 (23.03%)
No antibiotics	197 (18.45%)
Source of infection	
Respiratory tract	481 (45.04%)
Genitourinary	271 (25.37%)
Skin/Soft -Tissue	134 (12.55%)
GI tract/Liver	113 (10.58%)
Musculoskeletal	24 (2.25%)
Heart	7 (0.66%)
Intra-vascular catheters	3 (0.28%)
Blood	23 (2.15%)
Unknown	12 (1.12%)
Discharge disposition	
Home	528 (49.44%)
Nursing home	182 (17.04%)
Short term rehab	358 (33.52%)
Discharge hemoglobin	N=1064
	10.87±1.89 (10.75-10.98)
Peak WBC	N=1067
	16.78±7.83 (16.31-17.25)
Length of hospitalization	N=1066
	7.15±5.25 (6.84-7.47)
Duration of antibiotics	N=1067
	12.08±9.14 (11.52-12.64)
Categorical variables are presented as counts and percentages; continuous variables are presented according to their observed distribution (means and standard deviation).	

Among the total study population (1068), males aged >65 years formed a greater proportion. Four-hundred eighty (44.94%) participants were aged ≤65 years and 588 (55.06%) participants were aged >65 years; 567 (53.09%) participants were male while the remaining 501 (46.91%) participants were female. The majority of the participants presented from home, followed by the nursing home and short-term rehab. A large proportion of the patient population (44.85%) had previous hospitalizations in the past one year. The source of sepsis for the majority (45.04%) of the participants was respiratory tract infections, followed by the genito-urinary tract, skin/soft tissue, and gastrointestinal (GI) tract/liver, with 25.37%, 12.55%, and 10.58%, respectively. Bone/joints and blood were 2.25% and 2.15%, respectively. The majority of the patients were discharged home.

Hospital readmissions

A total of 205 (19.19%) patients were readmitted within 30 days. Of the 280 ICU patients, 72 (25.71%) patients were readmitted and of the 788 non-ICU patients, 133 (16.88%) patients were readmitted (Table 2). The majority of the readmissions across both populations was secondary to an infectious etiology (Table 3).

**Table 2:** Descriptive analysis of readmission in the study population (N=1068).

Readmission	Total	Non-ICU	ICU
Yes	205 (19.19%)	133 (16.88%)	72 (25.71%)

**Table 3:** Descriptive analysis of causes of readmission in the study population.

Cause of readmission	Total n=205
Infectious etiology	107 (52.20%)
Cardiovascular	26 (12.68%)
Gastrointestinal, hepatic and pancreatic diseases	21 (10.24%)
Musculoskeletal	12 (5.85%)
Respiratory system other than pneumonia	12 (5.85%)
CNS	8 (3.90%)
Genitourinary	8 (3.90%)
Hematological	3 (1.46%)
Psychiatric illness	2 (0.98%)
Neoplasm	2 (0.98%)
Opioid Overdose	2 (0.98%)
Alcohol withdrawal	1 (0.49%)
Lithium toxicity	1 (0.49%)

Factors associated with readmissions

The univariate logistic regression analysis identified associations between primary patient/hospitalization characteristics and 30-day readmissions (Table 4). The multivariate regression analysis was conducted for the factors, which showed a positive association on the univariate regression analysis (Table 5). After controlling for the effect of all the potential confounders, prior hospitalization in one year was significantly associated with readmission (adjusted odds ratio (AOR) 2.02, 95% CI 1.45 to 2.81, p-value <0.01). The other parameters that had shown a statistically significant association were the discharge disposition to short-term rehab (AOR 1.656, 95% CI 1.12 to 2.43, p-value 0.010) and nursing home (AOR 2.12, 95% CI 1.37 to 3.29, p-value 0.001), as compared with home. High discharge Hb was associated with a lower risk of readmission (AOR 0.842, 95% CI 0.75 to 0.93, p-value 0001). Length of stay, hemodialysis, duration of antibiotics, RBC transfusion, and ICU admission did not show an independent association with readmission.

**Table 4:** Univariate logistic regression analysis of factors associated with readmission in the study population. WBC: white blood cell, RBC: red blood cell; AKI: acute kidney injury; IV: intravenous, ICU: intensive care unit

Factor	Un-adjusted odds ratio	95 % CI of odds ratio	P value
Age (Base line= ≤65 years)			
>65 year	1.1350	0.987 – 1.842	0.059
Gender (Base line=Male)			
Female	1.020	0.752 – 1.384	0.897
Admission place (Base line= Home)			
Nursing home	1.861	1.301 - 2.661	0.001
Short-term rehab	1.691	0.775 – 3.687	0.187
Jail	1.628	0.168 – 15.769	0.749
Length of stay	1.049	1.022 – 1.076	<0.001
Hemodialysis (Base line=No)			
Yes	2.596	1.120 – 6.018	0.026
Prior hospitalization in 1 year (Base line=No)			
Yes	2.553	1.863 – 3.498	<0.001
Peak WBC	1.003	0.984 – 1.022	0.774
Source of infection (Base line= Lungs)			
Genito-Urinary Tract	0.760	0.517 – 1.117	0.162
GI tract/liver	0.652	0.386 – 1.100	0.109
Skin/Soft-Tissue	0.704	0.407 – 1.219	0.210
Bone/Joints	1.530	0.618 – 3.789	0.358
Blood	1.486	0.284 – 7.773	0.639
Heart	0.000	0.000	0.000
Intra-vascular catheters	1.032	0.374 – 2.538	0.951
Unknown	2.654	0.825 – 8.537	0.102
Discharge Disposition (Base line=Home)			
Short-Term Rehab	2.252	1.579 – 3.213	<0.001
Nursing Home	3.005	1.993 – 4.531	<0.001
Duration of Abx.	1.003	0.987 – 1.020	0.772
Discharge Hb	0.748	0.680 – 0.822	<0.001
Clostridium -difficile infection (Base line=No)			
Yes	1.636	0.867 – 3.087	0.128
RBC transfusions (Base line=No)			
Yes	0.494	0.310 – 0.789	0.003
Abx at discharge (Base line=NA)			
Oral	0.716	0.477 – 1.074	0.107
IV	1.366	0.875 – 2.134	0.170
ICU (Base line=No)			
Yes	1.705	1.230 – 2.363	0.001
AKI (Base line=No)			
Yes	1.163	0.819	1.652

**Table 5:** Multivariate logistic regression analysis of factors associated with readmission in the study population WBC: white blood cell, RBC: red blood cell; AKI: acute kidney injury; IV: intravenous, ICU: intensive care unit

Factor	Un-adjusted odds ratio	95 % CI of odds ratio	P value
Length of stay	1.015	0.982-1.049	0.371
Hemodialysis (Base line=No)			
Yes	1.945	0.803-4.712	0.141
Prior hospitalization in 1 year (Base line=No)			
Yes	2.020	1.450-2.814	<0.001
D/C Dispo (Base line=Home)			
Short-Term Rehab	1.656	1.127-2.433	.010
Nursing Home	2.126	1.374-3.291	.001
Duration of Abx	0.997	0.980-1.015	0.754
D/C Hb	.842	.758-.935	0.001
RBC transfusions (Base line=No)			
Yes	1.155	.687-1.941	0.586
ICU (Base line=No)			
Yes	1.017	.685-1.510	0.933

## Discussion

The cumulative rate of sepsis readmissions, regardless of the requirement of intensive care in our study, was 19.19%. The readmission rate in subjects requiring intensive care was 25.71%, and in non-ICU subjects, it was noted to be 16.88%. This concludes that readmissions after sepsis hospitalizations are common. Similar readmission rates have been observed in prior studies, but a wide variation exists, likely secondary to the variable case-mix [14-17]. Similar to prior studies, our study also depicted that the incidence of 30-day readmissions of sepsis patients is similar to the currently tracked four Centers for Medicare & Medicaid Services (CMS) conditions [18-19]. We hypothesized that admission to the intensive care unit will be associated with a higher readmissions rate, as patients requiring the intensive care unit are generally sicker and have severe sepsis. However, we did not find any statistically significant association of the requirement for intensive care with readmissions. Similar findings have been reported in studies done by Jones et al. and Gadre [13,18].

In our study, the most common cause of readmissions was infectious etiology. This finding is supported by similar studies where the infectious cause appeared to be the cause of readmissions in around 50% of the patients. Gadre et al. found that 42% of the readmissions were due to infectious causes [18]. Similarly, Chang et al. reported that infections accounted for 59.3% of the primary diagnosis on readmission at 30 days [20]. It has been hypothesized that increased readmissions from infectious causes could represent either an unresolved prior infection or the acquisition of a new infection stemming from immunosuppression caused by sepsis. Nosocomial infections like catheter-associated urinary tract infection (CAUTI) and central line-associated bloodstream infection (CLABSI) are also more common in sepsis patients and, therefore, the prompt removal of catheters and central lines in these patients can potentially reduce these readmissions. We also noticed that cardiovascular conditions like congestive heart failure, myocardial infarction, gastrointestinal diseases, and pulmonary diseases other than pneumonia also formed a major proportion of readmissions. Some of these readmissions can be prevented by early and close follow-up of the patients in outpatient settings [13,15,18].

We studied several factors in association with readmissions. We found that, in general, patients with sepsis who presented from nursing homes, hospitalized at least once in the past year, received hemodialysis, had a longer hospital stay, required the intensive care unit, had blood transfusions, or were discharged to either nursing homes or short-term rehabs had incurred higher readmission odds. However, of these factors, only prior hospitalization in one year and discharge disposition to nursing homes were statistically and independently associated with readmissions.

Various studies have established that readmission rates vary according to the discharge destination [15,17-18]. In an analysis done by Prescott, patients who were discharged to a nursing home either for the first time or who resided at nursing homes on a chronic basis formed a disproportionately large proportion of readmissions [15]. Our study found that patients who were long-term residents of a nursing home at the time of the index hospitalization or were discharged to nursing homes or short-term rehab suffered higher readmission rates. Improved communications with post-acute care providers and close follow-up can have some role in the prevention of these admissions. Length of stay has also been extensively studied in relation to hospital stay outcomes and a longer stay has been associated with an increased risk of readmissions in medical patients [21-22]. A longer length of stay is associated with an increased risk of nosocomial infections and adverse drug reactions, which ultimately increases the risk of readmission [18,23]. However, we did not see the same association in our study, likely because these patients either incurred high mortality or were transitioned to hospice because of poor prognosis. Similar to the study done by Sun et al., we found that prior hospitalizations in the past year were independently associated with higher readmissions [24]. The presence of past hospitalizations signify high co-morbidities, and this factor does not seem to be modifiable.

High hemoglobin showed a negative association with readmissions. Some studies have shown that packed RBC transfusion is associated with increased 30-day readmission [24]. However, restrictive transfusion practices and improved hemoglobin at discharge can prevent readmissions from symptomatic anemia or a worsening of underlying coronary artery disease (CAD).

Lastly, renal replacement therapy was also associated with an increased risk of readmissions, but the association was not statistically significant. AKI and the requirement of hemodialysis have been shown to be well-associated with high mortality as well as an increased risk of readmissions in septic patients [25].

## Conclusions

Our study concludes that readmissions after sepsis hospitalizations are common and mostly due to infectious causes. There are many factors associated with these readmissions, but a wide variation is noted across different studies. Only some of these factors are modifiable and not all hospital admissions are preventable. Some of the factors associated with readmissions are markers of the severity of illness rather than the target of intervention for readmission prevention. Nevertheless, readmissions after sepsis are common, and more research should be done to identify clear targets of intervention.
